# COVID-19 vaccine hesitancy in Sweden and Italy: The role of trust in authorities

**DOI:** 10.1177/14034948221099410

**Published:** 2022-06-02

**Authors:** Elena Raffetti, Elena Mondino, Giuliano Di Baldassarre

**Affiliations:** 1 Centre of Natural Hazards and Disaster Science, Sweden; 2 Department of Earth Sciences, Uppsala University, Sweden; 3 Department of Public Health and Primary Care, Cambridge University, UK; 4 Department of Global Public Health, Karolinska Institutet, Sweden

**Keywords:** COVID-19, vaccination, preventive medicine, health policy

## Abstract

**Background:**

The success of vaccination campaigns against COVID-19 infection is vital for moving from a COVID-19 pandemic to an endemic scenario. We aimed to unravel the influence of the risk perception of epidemics along with individual and contextual factors on adherence to COVID-19 vaccination campaigns in Italy and Sweden.

**Methods:**

We compared the results of two nationwide surveys carried out in August 2021 across four domains of epidemic risk perception: perceived likelihood, perceived impact on the individual and perceived individual and authority knowledge. The roles of individual and contextual determinants were also explored.

**Results:**

The survey included 2144 participants in Sweden (52.3% women) and 2010 in Italy (52.6% women). In both countries, we found that trust in authorities was one of the main drivers of this process, with two-fold increased odds of being vaccinated. Being highly educated and having a higher relative income were associated with a higher adherence to the vaccination campaign (for relative income OR = 1.44, 95% CI 1.23–1.67 in Sweden and OR = 1.18, 95% CI 1.04–1.34 in Italy; for education OR = 1.90, 95% CI 1.30–2.77 in Sweden and OR = 1.47, 95% CI 1.09–1.97 in Italy), whereas a right and centre-right compared with a left and centre-left political orientation was negatively related to vaccination adherence (OR = 0.41, 95% CI 0.25–0.67 in Sweden and OR = 0.47, 95% CI 0.33–0.68 in Italy).

**Conclusions:**

Increasing trust in authorities, along with an equal global distribution of vaccine doses, can contribute to accelerating vaccination campaigns around the world and, in turn, to move towards an endemic scenario.

## Introduction

The COVID-19 pandemic has led to unprecedented medical, economic and social consequences. Although a wide range of potential treatments has been tested for COVID-19, efficacy and access remain low [1]. There is a near-universal consensus: enough of the population should be immunized to prevent the exponential growth of new cases and alleviate the adverse effects in case of contagion [2]. At present, vaccination appears to be the most advantageous strategy towards this goal. Successful vaccination programmes rely on three main factors: timeliness of vaccine development; people’s willingness to be vaccinated; and equal distribution [3]. In terms of vaccine development, in less than one year from the beginning of the COVID-19 pandemic, we have faced unparalleled vaccine production worldwide: six vaccines have currently been approved by >50 countries [4]. Current research frontiers assure safe and effective vaccines in reducing the risk of COVID-19 infection, severe illness and the spread of disease [5]. A prominent example is the 90% efficacy of mRNA vaccines in preventing severe cases, also associated with new SARS-CoV-2 variants, during the first six months from the second dose [6].

Vaccination programmes are now facing a crucial aspect: people’s willingness to be vaccinated. Recent surveys have shown that a proportion of the population in many countries is unwilling to be vaccinated [7,8]. Vaccine acceptance stems from the perception of vaccination safety, risk of disease and trust in authority. Despite much we have learned from past vaccination programmes, this campaign has unique characteristics. The rapid pace of vaccine development encompasses the use of new technology, which, along with emergency use authorizations for vaccines to prevent COVID-19 infection [9], may lead to a belief of lower vaccine safety and a higher risk of unexpected side effects. Although the dramatic experience of the pandemic may encourage adherence to the vaccination campaign, one year of strict measures that affect personal freedom have led to, or exacerbated, the mistrust of governmental authorities in many countries [10]. This fits into a global scenario of growing anti-vaccination movements during the last 20 years, characterized by misinformation and non-acceptance of the safe and long-term use of vaccines [11].

At odds with anti-vaccination movements, a low willingness to be vaccinated against COVID-19 infection reflects more complex concerns [12]. Individuals base their choices on precautionary principles: what is the minimum harm? Theoretically, an individual may examine two probabilities: the probability of being infected with COVID-19 and a severe complication, and the probability of being vaccinated and suffering from a side effect. Along with this, individuals may weigh the severity of possible effects associated with the vaccine and COVID-19 infection to a different extent. How such processes unfold clearly varies among individuals and may be influenced by the above-mentioned factors, above all trust in authority. Other mechanisms may contribute to exacerbate this process, such as optimistic bias, media propagation, fear of unstated severe side effects, along with confirmation of concerns – for example, the rare blood clots in unusual sites associated with thrombocytopenia after COVID-19 vaccination with viral vector vaccines [13].

Globally, adherence to vaccination campaigns can contribute to achieve the goal of reducing the risk of COVID-19 outbreaks. However, the world will move from a pandemic to an endemic phase only if high-income countries support an equal global distribution of vaccine doses [14]. Despite 4.17 billion doses being administered up to 1 August 2021 [15], direct purchase agreements have allowed high- and middle-income countries to pre-order large numbers of doses and speed up their vaccination campaigns, while low-income countries can only rely on the World Health Organization COVAX programmes [16].

Understanding the underlying (individual and societal) determinants of vaccine adherence is important in achieving high rates of vaccination against COVID-19 globally. Although individual determinants have been widely studied [7,8] and are currently considered in vaccination planning and management, the risk perception of epidemics should also be considered when organizing communication campaigns and community involvement. Here, we aim to unravel the relationship between the public perceptions of epidemic risks and adherence to vaccination campaigns against COVID-19. We focused on Italy and Sweden: two countries in the European Union with similarities in their welfare state organization, but with differences in both the authority response to the COVID-19 pandemic [17] and their history of public scepticism about vaccination campaigns [18]. We first evaluated the role of public perceptions of epidemic risks and individual determinants in adherence to the COVID-19 vaccination campaign. We then examined the role of contextual determinants in Italy and Sweden.

## Methods

A population-based anonymous survey of public risk perception was carried out in Italy and Sweden between 13 and 23 August 2021. The samples were representative of the Swedish and Italian population for sex and age and were derived from two existing national survey panels (about 100,000 participants) in each country established by the marketing research company Kantar Sifo. In brief, the sample was derived as a random sample among panellists and weights (based on sex, age and region) were applied to ensure that the results were representative of the population. The geographical distribution of the sample was representative of the geographical distribution of the population by region in both countries, with over-representation of the capital region populations, with a 1/9 sampling ratio in Italy and a 4/6 sampling ratio in Sweden. The overall participation rate was 27.5%. The survey explored the public risk perception for nine global threats: epidemics, droughts, flooding, wildfires, air pollution, earthquakes, economic crisis, domestic violence and terror attacks.

This analytical sample included 2144 participants in Sweden (52.3% women) and 2010 in Italy (52.6% women). We considered the public epidemics perception of four domains – likelihood, impact on the individual, and individual and authority knowledge – using a Likert-type scale (from a minimum of 1 to a maximum of 5). Perceived authority knowledge was considered as a proxy of the trust in authorities. Each item was standardized for the risk perception of the other threats (e.g. perceived epidemics likelihood/mean perceived likelihood for the other threats). Vaccination acceptance was defined as self-reported vaccination against COVID-19 with at least one dose. Information on direct experience of an epidemic (yes/no, non-limited only to the COVID-19 pandemic) and socioeconomic factors, such as employment (yes/no), relative income, university education (yes/no), political orientation (left, centre-left, centre, centre-right, right) were considered as possible individual-level determinants of vaccination acceptance.

The prevalence of vaccination against COVID-19 with at least one dose at the regional level was retrieved from the Swedish and Italian National Institute for Health database at the time of the survey [19,20]. Cumulative incidence of COVID-19 cases per 1000 individuals [21] at the time of the survey and excess mortality during the first wave (15 February–15 May 2020 for Italy [22] and 1 March–31 May 2020 for Sweden) at the regional level (Nomenclature of Territorial Units for Statistics 2 Classification of the European Union ) were encompassed as country-level determinants.

Descriptive statistics were used to summarize the main characteristics of the study sample. The associations between domains of epidemics risk perception and vaccination acceptance were assessed using logistic regression models. The results were expressed in terms of odds ratios (ORs) and corresponding 95% confidence intervals (CIs). The role of country as an effect modifier was examined with formal interactions. Bivariable and multivariable logistic regression models were used to examine the association between individual-level factors – such as epidemic experience, employment, relative income, education and political orientation – as possible determinants of vaccination acceptance (independent variables). Adjustment for age was applied to all models because a vaccine against COVID-19 was offered to all age groups for adults only from June 2021.

We ran an ecological analysis to examine if prevalence of vaccination against COVID-19 with at least one dose varied according to the extent to which an area was affected by the COVID-19 pandemic using linear regression models stratified by country.

Some limitations should be kept in mind. Although national samples should be considered representative of the general populations in terms of age and sex [23], an over-representation of the population with a non-immigration background was expected in both countries due to linguistic barriers. However, we could not compare the participation rate in individuals with and without an immigrant background because this information was not collected. This may have resulted on a higher vaccination rate in the analytical sample compared with the general population. The specific role of restrictive measures and media coverage as possible determinants of vaccination adherence could not be evaluated with this data. Perceived authority knowledge measured only one domain of the trust in authorities.

## Results

### Epidemics risk perception, individual determinants and vaccination adherence

The sample included 2144 individuals in Sweden (52.3% women) and 2010 in Italy (52.6% women). The prevalence of vaccination against COVID-19 infection with at least one dose was 93.5% in Sweden and 85.3% in Italy. The proportion of individuals with employment and university education was higher in Sweden than in Italy (see Supplementary Table 1, available online).

**Figure 1.**
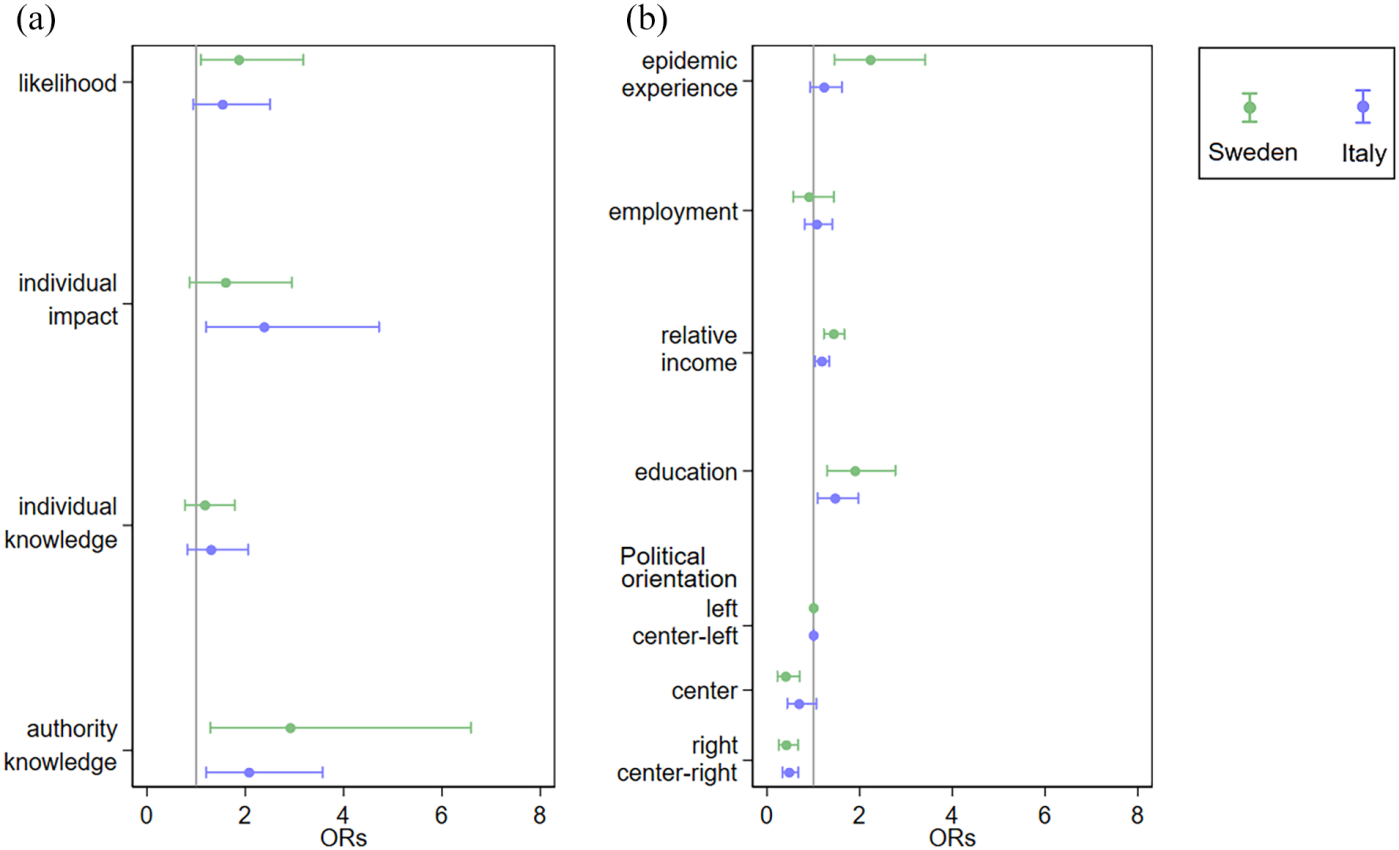
Association of (a) epidemic risk perception and (b) individual determinants with adherence to vaccination programme against COVID-19 infection. The results should interpreted in terms of the odds ratio with 1 as the null value (grey lines).

Figure 1(a) shows the relation between COVID-19 vaccination adherence and four domains of epidemic risk perception (perceived likelihood, perceived individual impact, and perceived individual and authority knowledge) derived from our population-based survey (see Supplementary Table 2, available online, for the magnitude of ORs). Overall, the perception of authorities’, but not individuals’, knowledge was associated with a two-fold increased odds of being vaccinated. In Sweden, there was a positive association between perceived likelihood of epidemics and adherence to the vaccination programme, whereas in Italy the impact of the epidemics was positively related to COVID-19 vaccination.

Figure 1(b) shows how COVID-19 vaccination adherence was associated with specific individual determinants: epidemic experience, employment, relative income, education and political orientation (see Supplementary Table 3, available online, for the magnitude of ORs). High relative income and university education compared with low income and low level of education gave greater odds of being vaccinated (for relative income OR = 1.44, 95% CI 1.23–1.67 in Sweden and OR = 1.18, 95% CI 1.04–1.34 in Italy; for education OR = 1.90, 95% CI 1.30–2.77 in Sweden and OR = 1.47, 95% CI 1.09–1.97 in Italy). Political orientation also influenced vaccination coverage, with right- and centre-right-oriented individuals less adherent to the vaccination campaign than centre-left- and left-oriented individuals (OR= 0.41, 95% CI 0.25–0.67 in Sweden and OR= 0.47, 95% CI 0.33–0.68 in Italy). Of note, experience of epidemics was associated with a two-fold higher likelihood of being vaccinated only in Sweden (Figure 1(b)). A similar pattern of associations and similar magnitude of ORs was found in fully adjusted multivariable models (see Supplementary Table 3 and Supplementary Figure 1, available online).

### Contextual factors and prevalence of vaccination with at least one dose

**Figure 2.**
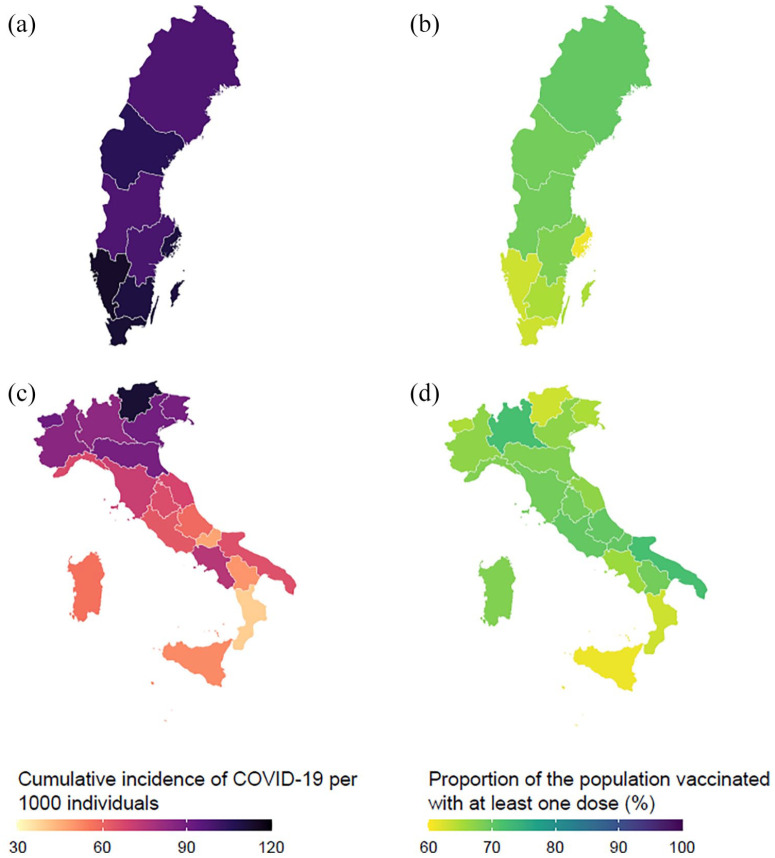
Cumulative incidence of COVID-19 cases per 1000 individuals for (a) Sweden and (c) Italy. Proportion of population vaccinated against COVID-19 with at least one dose for (b) Sweden and (d) Italy bottom. Data for mid-August 2021, stratified by region.

We analysed national surveillance data to evaluate the role of contextual factors. Figure 2 shows the cumulative number of total COVID-19 cases per 1000 individuals and the proportion of the population vaccinated against COVID-19 in Italy and Sweden at the time of the survey (mid-August 2021). Strikingly, although the distribution of cases in Sweden followed a geographical gradient from south to north, the proportion of the population vaccinated was in the opposite direction, from north to south. In the Italian context, the COVID-19 cumulative incidence had a well-defined geographical north–south pattern, but there was no clear pattern in the distribution of vaccination coverage. A formal analysis using linear regression models confirmed an inverse association between COVID-19 cumulative infection rate and the proportion of population vaccinated in Sweden (coefficient −0.42, 95% CI −0.67 to −0.17) and no association in Italy (coefficient −0.03, 95% CI −0.11 to 0.05). Excess mortality at the regional level was slightly associated with a lower vaccination adherence in the Swedish context (coefficient −0.11, 95% CI −0.20 to −0.01), whereas no association was found in the Italian context (coefficient 0.01, 95% CI −0.05 to 0.06).

## Discussion

Advancing knowledge about the influence of epidemics risk perception, along with individual and contextual factors on vaccination adherence, is fundamental for moving from a pandemic to an endemic global scenario. Here, we show that trust in authority (measured as authority knowledge) and political orientation were among the main drivers of vaccination adherence, in particular in a context as Italy, characterized by a robust authority response to the pandemic. With respect to epidemic risk perception, we found that trust in authority drives vaccination adherence more than the risk of disease. Perception of trust in authority was strongly associated with adherence to vaccination campaigns in both countries, whereas a relation with the perceived likelihood of being affected was only observed in the Swedish context. This might be explained by two concurrent phenomena. Summer months are characterized by a low incidence of COVID-19 infection and plausibly by a lower risk perception of likelihood of the epidemic [24]. Along with this, reduced trust in authority may conceal the part played by the perception of disease severity and may discourage individuals from getting vaccinated. It is worth noting that this pattern mainly occurred in the Italian context. The Italian government’s response, also during the vaccination campaign, has mainly been driven by decrees and mandatory restrictive measures, whereas the Swedish response has been primarily based on recommendations [17]. This is also reflected by the average of the national stringency index, which measures COVID-19 national responses (0–100), which was higher in Italy than in Sweden (67.5 versus 55.4) [25].

We also found that those who were more educated and had a higher income were more adherent to the COVID-19 vaccination campaign. Our study shows that individuals with centre-right/right political orientations had a lower coverage for COVID-19 vaccination than individuals with a centre-left political orientation. This can be plausibly explained by the political orientation of the current Swedish and Italian governments – that is, centre-left in Sweden and a national unity government with the right-wing as the main party in opposition in Italy. A diverging political orientation from the national government may plausibly influence the perception of trust in authority. This may be exacerbated by several months of imposed and recommended restrictive measures [25], along with historical reasons in the Swedish context. Sweden has a tradition of a top-down consensus culture with a high trust in authority and compliance [26].

By comparing the results between countries, this study also contributes to understanding the role of contextual factors. A lower vaccination rate in the Swedish regions with a higher cumulative incidence of COVID-19 may be explained by doctors’ recommendation of waiting few months from the date of infection before being vaccinated for individuals with a history of COVID-19 infection [27]. Along with this, overall low adherence to authority’s recommendations may, in turn, influence both COVID-19 spread and vaccination. The three regions with the lowest vaccination coverage are those with the most populated cities (Stockholm, Gothenburg and Malmo), and the highest proportion of immigrant population [28]. Recent reports confirm the hypothesis that some low-income urban areas with a high proportion of immigrant population in Stockholm, Gothenburg and Malmo are characterized by a high cumulative incidence of COVID-19 infection and low vaccination coverage [29]. However, a lack of proper communication about the risk related to COVID-19 and vaccine safety, and limited community involvement, may negatively influence trust in authority and, in turn, compliance with recommendations in some minority groups. A proportion of immigrant groups are not completely integrated into the Swedish consensus culture: they live segregated from the majority of the Swedish population and are influenced from their home country rather than Swedish media.

A larger population at the regional level and a different immigration pattern prevented a comparison of the Swedish and Italian contexts. Although there was no clear association between cumulative incidence of COVID-19 infection and vaccination coverage in the Italian context, four of the five Italian regions with the lowest vaccination coverage are autonomous (i.e. they are more independent from the central government in certain aspects of their administration). Along with a self-directed organization of the vaccination campaign, the population of the autonomous regions may mistrust central government as a result of historical reasons.

Despite massive efforts to produce and administer vaccines, a proportion of the population is not willing to be vaccinated, even in countries with available vaccines. We found that trust in authority was one of the main drivers of this process. Whether and how governments and public health institutes direct efforts to improve trust in public authorities is therefore pivotal to increasing COVID-19 vaccination coverage and, in turn, moving from a pandemic to a global endemic scenario.

## Supplemental Material

sj-docx-1-sjp-10.1177_14034948221099410 – Supplemental material for COVID-19 vaccine hesitancy in Sweden and Italy: The role of trust in authorities Supplemental material, sj-docx-1-sjp-10.1177_14034948221099410 for COVID-19 vaccine hesitancy in Sweden and Italy: The role of trust in authorities by Elena Raffetti, Elena Mondino and Giuliano Di Baldassarre in Scandinavian Journal of Public Health

sj-docx-2-sjp-10.1177_14034948221099410 – Supplemental material for COVID-19 vaccine hesitancy in Sweden and Italy: The role of trust in authorities Supplemental material, sj-docx-2-sjp-10.1177_14034948221099410 for COVID-19 vaccine hesitancy in Sweden and Italy: The role of trust in authorities by Elena Raffetti, Elena Mondino and Giuliano Di Baldassarre in Scandinavian Journal of Public Health
