# Supplementary material for: COVID-19 vaccine hesitancy in Sweden and Italy: The role of trust in authorities
Source: Scand J Public Health. 2022 Jun 2;50(6):803–9. doi: 10.1177/14034948221099410 (PMC9361415; doi:10.1177/14034948221099410)
Supplement: sj-docx-1-sjp-10.1177_14034948221099410 – Supplemental material for COVID-19 vaccine hesitancy in Sweden and Italy: The role of trust in authorities [file sj-docx-1-sjp-10.1177_14034948221099410.docx]

**Supplementary table 1.** Participants’ characteristics by country.

|  | **Sweden**  **n (%)** | **Italy**  **n (%)** |
| --- | --- | --- |
| Total | 2144 | 2010 |
| Females | 1121 (52.3) | 1057 /52.6) |
| Age, mean (SD) | 49.4 (16.6) | 49.1 (14.8) |
| At least one dose of vaccination against COVID-19 infection | 1986 (93.5) | 1633 (85.3) |
| Employment | 1467 (69.0) | 1164 (59.0) |
| University education | 1330 (62.5) | 711 (35.5) |
| Political orientation |  |  |
| Left- center left | 776 (37.9) | 688 (44.4) |
| Center | 346 (16.9) | 275 (17.7) |
| Right-center right | 926 (45.2) | 588 (37.9) |

**Supplementary table 2**. ORs and 95% CIs for the association between epidemics risk perception and adherence to vaccination program against COVID-19 infection.

|  | **Sweden** | **Italy** |
| --- | --- | --- |
|  | **ORs (95% CIs)** | **ORs (95% CIs)** |
| Likelihood | 1.87 (1.10,3.18) | 1.54 (0.94,2.50) |
| Impact on the individual | 1.60 (0.87,2.95) | 2.38 (1.20,4.72) |
| Individual knowledge | 1.18 (0.77,1.79) | 1.30 (0.82,2.06) |
| Authority knowledge | 2.91 (1.29,6.59) | 2.07 (1.20,3.57) |

**Supplementary table 3**. ORs and 95% CIs for the association between individual determinants and adherence to vaccination program against COVID-19 infection.

|  | **Sweden** |  |  | **Italy** |  |
| --- | --- | --- | --- | --- | --- |
|  | **Adjusted for age** | **Fully adjusted** |  | **Adjusted for age** | **Fully adjusted** |
|  | **ORs (95% CIs)** | **ORs (95% CIs)** |  | **ORs (95% CIs)** | **ORs (95% CIs)** |
| Epidemic experience | 2.23 (1.46,3.41) | 1.98 (1.23,3.17) |  | 1.23 (0.93,1.62) | 1.23 (0.89,1.71) |
| Employment | 0.90 (0.57,1.44) | 0.77 (0.45,1.33) |  | 1.07 (0.81,1.41) | 0.96 (0.68,1.36) |
| Relative income | 1.44 (1.23,1.67) | 1.51 (1.25,1.82) |  | 1.18 (1.04,1.34) | 1.20 (1.03,1.40) |
| Education | 1.90 (1.30,2.77) | 1.81 (1.14,2.88) |  | 1.47 (1.09,1.97) | 1.13 (0.80,1.59) |
| Political orientation |  |  |  |  |  |
| Left-center left | 1.00 | 1.00 |  | 1.00 | 1.00 |
| center | 0.40 (0.23,0.71) | 0.49 (0.26,0.92) |  | 0.69 (0.44,1.07) | 0.69 (0.44,1.07) |
| Right-center right | 0.41 (0.25,0.67) | 0.42 (0.25,0.72) |  | 0.47 (0.33,0.68) | 0.50 (0.35,0.73) |


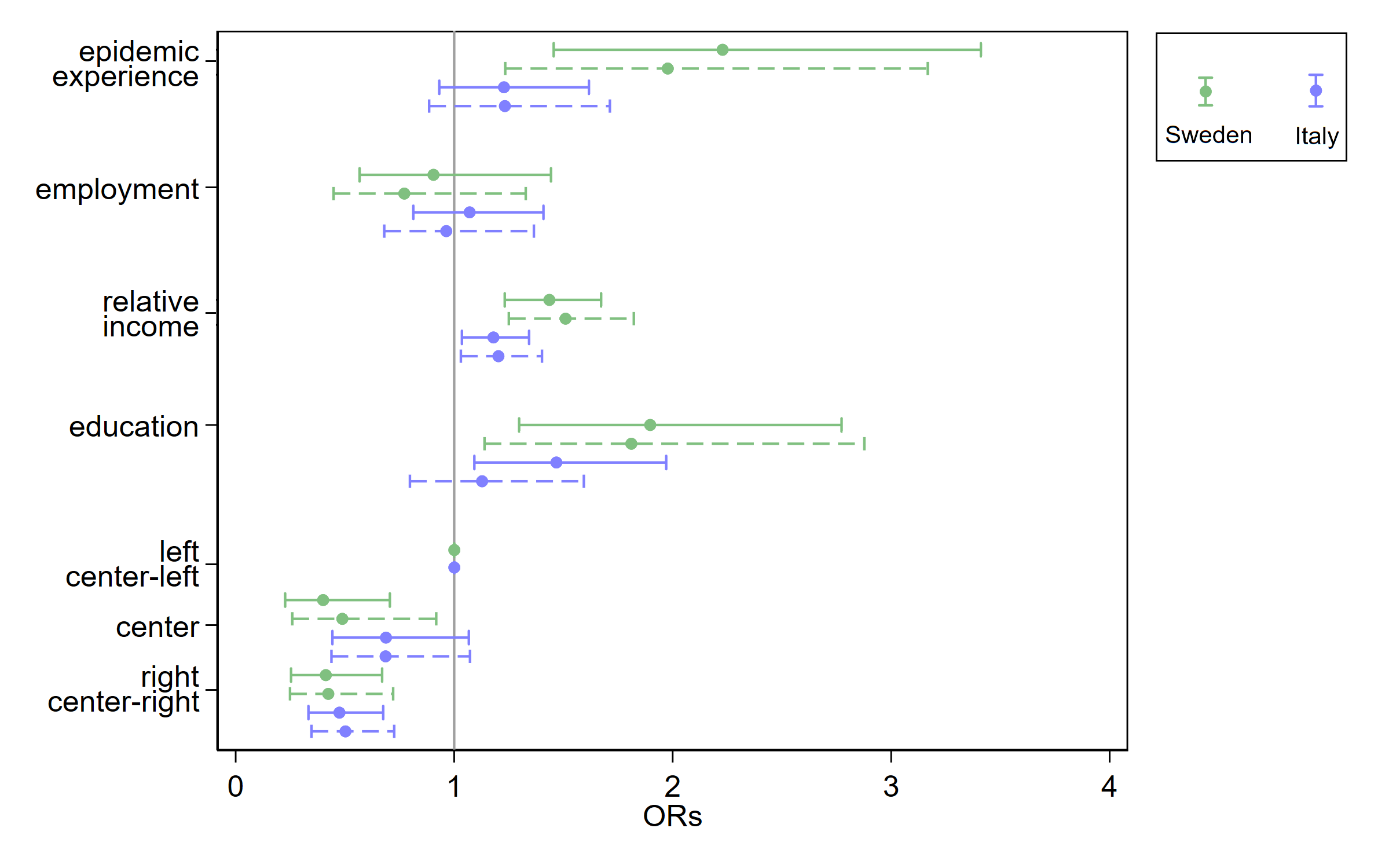


**Supplementary figure 1**. Adjusted for age (continuous line as in Figure 1) and fully adjusted (dashed line) ORs and 95% CIs for the association of individual determinants and adherence to vaccination program against COVID-19 infection.
